# Fisetin stimulates autophagic degradation of phosphorylated tau via the activation of TFEB and Nrf2 transcription factors

**DOI:** 10.1038/srep24933

**Published:** 2016-04-26

**Authors:** Sunhyo Kim, Ki Ju Choi, Sun-Jung Cho, Sang-Moon Yun, Jae-Pil Jeon, Young Ho Koh, Jihyun Song, Gail V. W. Johnson, Chulman Jo

**Affiliations:** 1 Division of Brain Diseases, Center for Biomedical Sciences, Cheongju-si, Chungcheongbuk-do 363-951, Korea; 2 Division of Biosafety Evaluation and Control, Korea National Institute of Health, 187 Osongsaengmyeong2(i)-ro, Osong-eup, Cheongju-si, Chungcheongbuk-do 363-951, Korea; 3 Department of Anesthesiology, University of Rochester Medical Center, University of Rochester, 601 Elmwood Ave., Rochester, NY 14642, USA

## Abstract

The neuronal accumulation of phosphorylated tau plays a critical role in the pathogenesis of Alzheimer’s disease (AD). Here, we examined the effect of fisetin, a flavonol, on tau levels. Treatment of cortical cells or primary neurons with fisetin resulted in significant decreases in the levels of phosphorylated tau. In addition, fisetin decreased the levels of sarkosyl-insoluble tau in an active GSK-3β-induced tau aggregation model. However, there was no difference in activities of tau kinases and phosphatases such as protein phosphatase 2A, irrespective of fisetin treatment. Fisetin activated autophagy together with the activation of transcription factor EB (TFEB) and Nrf2 transcriptional factors. The activation of autophagy including TFEB is likely due to fisetin-mediated mammalian target of rapamycin complex 1 (mTORC1) inhibition, since the phosphorylation levels of p70S6 kinase and 4E-BP1 were decreased in the presence of fisetin. Indeed, fisetin-induced phosphorylated tau degradation was attenuated by chemical inhibitors of the autophagy-lysosome pathway. Together the results indicate that fisetin reduces levels of phosphorylated tau through the autophagy pathway activated by TFEB and Nrf2. Our result suggests fisetin should be evaluated further as a potential preventive and therapeutic drug candidate for AD.

Alzheimer’s disease (AD), the most common neurodegenerative disease in the elderly, is characterized by the presence of extracellular amyloid plaques and intracellular neurofibrillary tangles (NFTs) composed of hyperphosphorylated tau in the brain^1^. NFT pathology clinically correlates with dementia even better than amyloid pathology^2^. Recent studies have provided compelling evidence that phosphorylated tau plays a crucial role as a mediator of Aβ toxicity, thus compromising neuronal dysfunction and death^3^,^4^,^5^. Given these findings, there is a growing interest in finding molecules which are able to increase the clearance of tau in neurons as a therapeutic strategy for treating AD^6^.

Fisetin (3,7,3′,4′-tetrahydroxyflavone) is an organic flavonoid present in numerous fruits and vegetables such as strawberries, mangoes and cucumbers. Originally, it was identified in a screen of flavonoids which could prevent oxidative stress-mediated neuronal cell death^7^. In a study where approximately 30 flavonoids were evaluated for their ability to induce neuro-differentiation in PC12 cells, fisetin showed neurotrophic activity distinct from other flavonoids, and exhibited the most potent, neuroprotective effects^8^. Fisetin facilitates long-term potentiation in hippocampal slices, and promotes memory in wild type mice^9^. Fisetin also has a strong anti-inflammatory activity in brain^10^,^11^, and its oral administration significantly attenuated the development of learning and memory deficits in an AD mouse model^12^. Together, these findings strongly indicate that fisetin is a small, orally active molecule with a variety of biological activities that could likely attenuate AD pathology.

Autophagy is a series of intracellular membrane trafficking events involved in the organized elimination of proteins, organelles and invading microbes by lysosomes. For efficient clearance of sequestered contents, autophagy requires enhanced lysosomal activity as well as its induction. Recently, the transcriptional factor EB (TFEB) was reported to be a master regulator coordinating the expression of autophagy and lysosomal genes, and stimulating lysosomal biogenesis^13^,^14^. In normal conditions, TFEB is phosphorylated by mammalian target of rapamycin complex 1 (mTORC1) which localizes on the lysosomal surface, and thus is maintained in the cytoplasm^15^. When a cell is stressed or starved mTORC1 is inactivated via the amino acid/Rag GTPase pathway preventing TFEB phosphorylation and thus allowing it to move into the nucleus, where it induces downstream target genes such as ATG9b, p62/sequestosome (SQSTM) 1 and LAMP1 by binding to the coordinated lysosomal expression and regulation (CLEAR) element^13^,^15^. Of note, a recent study showed that TFEB promotes the clearance of aberrant tau species and rescues behavioral and synaptic deficits in a tauopathy mouse model^16^.

Growing evidence suggests that tau is mainly cleared by autophagy^17^,^18^,^19^,^20^. Recently, a study showed that autophagic dysfunction in AD model mice is enhanced by deletion of nuclear factor E2-related factor 2 (Nrf2)^21^. Moreover, our group provided evidence that the transcriptional activity of Nrf2 is essential for the clearance of phosphorylated tau via the selective autophagy^18^. Interestingly, fisetin not only activates Nrf2^22^, but also inhibits the activity of mTOR kinase^23^,^24^. Thus, we hypothesized that fisetin could promote the degradation of phosphorylated tau by enhancing autophagy through increasing the transcriptional activity of TFEB and Nrf2. In this study we show that fisetin facilitates the degradation of phosphorylated tau and provide evidence of the molecular mechanisms involved. Our results strongly suggest fisetin could be a therapeutic drug candidate for AD.

## Results

### Fisetin reduces levels of phosphorylated tau

**Figure 1:**
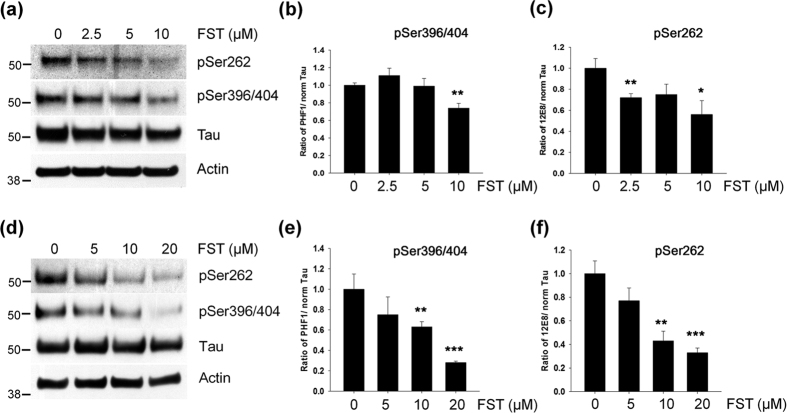
Fisetin reduces the levels of phosphorylated tau. (**a–c**) Mouse cortical neuronal cells (T4) were maintained in the presence of doxycycline (1 μg/ml) to induce the expression of tau for 12 h, and subsequently treated with different concentrations of fisetin (FST) for 24 h. (**d–f**) Rat primary cortical neurons (DIV10) were treated with different concentration of fisetin (FST) for 36 h. (**a,d**) The levels of tau phosphorylated at Ser262 and Ser396/404 and total tau were analyzed by immunoblotting using the 12E8, PHF1 and tau antibodies, respectively. (**b,c,e,f**) Bar graphs represent the relative ratio of phosphorylated tau to the level of tau normalized with that of actin. Data shown are mean ± SE of three independent experiments and were analyzed using Student’s *t* test. (*p < 0.05; **p < 0.01; ***p < 0.001).

To examine whether fisetin could affect phosphorylated tau levels, mouse cortical neuronal cells (T4) that inducibly express wild-type tau were treated with several concentrations of fisetin. Twenty-four hours after treatment with 10 μM of fisetin the levels of tau phosphorylated at Ser262 (12E8) and at S396/S404 (PHF1) were significantly reduced in mouse cortical cells (Fig. 1a–c). Significant decreases in the levels of phosphorylated tau were also observed in rat primary cortical neurons (DIV 10) following treatment with 10 or 20 μM of fisetin for 36 h (Fig. 1d–f). Of note, the fisetin-induced decrease in phosphorylated tau levels was concentration-dependent in both mouse cortical cells and primary neurons (Fig. 1). Treatment of mouse cortical cells with 5 or 10 μM fisetin and primary cortical neurons with 10 or 20 μM fisetin did not significantly alter viability (Supplementary Fig. S1). These results clearly indicate that the reduction of phosphorylated tau was not due to increased cell death. Thus, these results suggest that fisetin facilitates the degradation of phosphorylated tau in neurons.

### Fisetin reduces sarkosyl-insoluble tau

**Figure 2:**
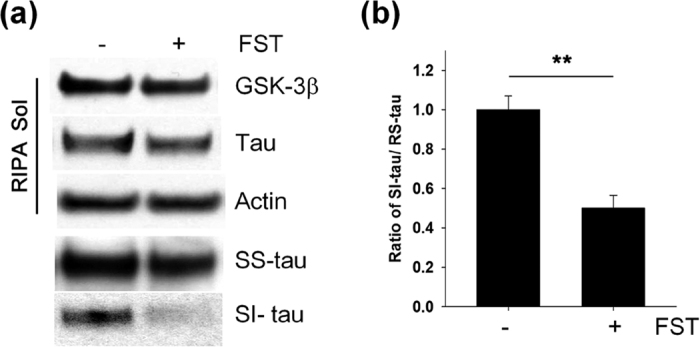
Fisetin reduces the levels of sarkosyl-insoluble tau. HEK 293 cells were co-transfected with plasmids expressing tau and constitutively active HA-GSK-3β-S9A, and subsequently treated with 10 μM fisetin (FST) for 24 h. RIPA-soluble (RS), sarkosyl-soluble (SS) and sarkosyl-insoluble (SI) fractions from the cells were prepared according to the protocol described in Methods. (**a**) The levels of tau and HA-GSK-3β-S9A in the cellular fractions were analyzed by immunoblotting using the total tau and HA antibodies, respectively. (**b**) Bar graphs represent the relative ratio of sarkosyl-insoluble tau to the level of RIPA-soluble tau normalized with that of actin. Data shown are mean ± SE of three independent experiments and were analyzed using Student’s *t* test. (**p < 0.01).

To examine whether fisetin could affect sarkosyl-insoluble tau levels, we set a cellular system co-transfected with plasmids expressing tau and constitutively active GSK-3β-S9A in which sarkosyl-insoluble tau was previously observed^25^,^26^. HEK 293 cells co-transfected with the plasmids were treated with 10 μM of fisetin. Twenty-four hours after treatment with fisetin the levels of sarkosyl-insoluble tau were significantly reduced in the HEK 293 cells (Fig. 2). Thus, these results suggest that fisetin inhibits the formation of sarkosyl-insoluble tau.

### Fisetin does not affect activities of tau kinases or phosphatases

**Figure 3:**
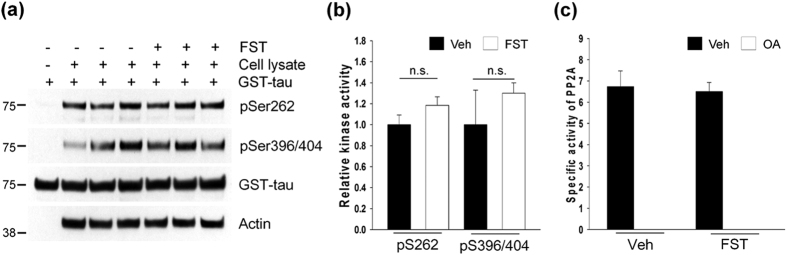
There is no significant change in tau kinase and phosphatase activities by fisetin treatment. (**a**) Mouse cortical cells (T4) were treated with either DMSO (Veh) or 5 μM fisetin (FST) for 12 h. Tau kinase assay was performed according to Methods. The levels of tau phosphorylated at Ser262 and Ser396/Ser404 were analyzed by immunoblotting using a 12E8- and PHF1-specific antibody, respectively. GST-tau was detected with a total tau antibody. (**b**) Bar graph of the relative optical density of phosphorylated tau normalized to actin. Data shown are mean ± SE and were analyzed using Student’s *t* test. (**c**) The phosphatase activity of PP2A in cell lysates was quantitated using the Serine/Threonine Phosphatase Assay System (Promega) by measuring the dephosphorylation of a phospho-peptide, RRA(pT)VA in the presence or absence of okadaic acid (OA, 20 nM). Data shown are mean ± SE of three independent experiments and were analyzed using Student’s *t* test. n.s. means data are not significant.

Tau has been suggested to be phosphorylated by several kinases such as glycogen synthase kinase-3β (GSK-3β), cyclin-dependent kinase 5 (Cdk5), extracellular signal regulated kinase 2 (ERK2), and microtubule-affinity regulating kinases (MARKs)^27^. Given the results, it is possible that fisetin-induced decreases in the levels of phosphorylated tau could result from lowered tau kinase activity in cells exposed on fisetin. Thus, we performed *in vitro* kinase assays using lysates from cortical cells incubated in the absence or presence of fisetin. These results showed that there was no significant difference in their kinase activities, irrespective of fisetin treatment (Fig. 3a,b). It also can be speculated that in cells treated with fisetin there may be increased activity of protein phosphatases such as protein phosphatase 2A (PP2A), the primary phosphatase involved in tau dephosphorylation^28^,^29^. When the activity of PP2A was assayed using cortical cell lysates, there was no difference in activities between cells treated with fisetin or not (Fig. 3c). In addition, there was no significant difference in the levels of phosphorylated GSK-3β and ERK1/2 by an extended time point 48 h (Supplementary Fig. S2). These results strongly support the conclusion that the decreased levels of phosphorylated tau in fisetin-treated neurons do not result from alterations in the activities of tau kinases or phosphatases.

### Fisetin activates TFEB

**Figure 4:**
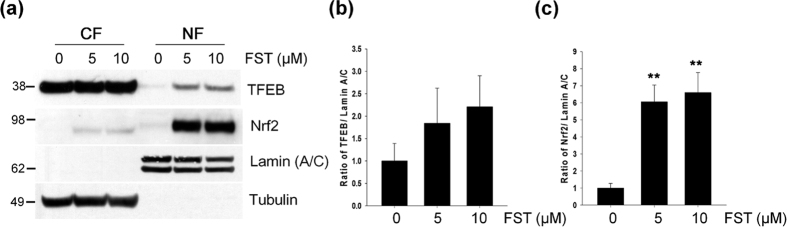
Fisetin induces the nuclear localization of TFEB and Nrf2. Mouse cortical cells (T4) were treated with either DMSO (Veh) or 5 μM fisetin (FST) for 6 h. (**a**) Nuclear (NF) and cytosolic (CF) fractions from the cells were prepared according to the procedure described in Methods. The cellular localization of TFEB and Nrf2 was analyzed by immunoblotting using the anti-TFEB and Nrf2 antibodies, respectively. To examine the purity of the fractionations, the blot was probed with antibodies to lamin (A/C), a marker for nuclear fraction, and tubulin, a marker for cytosolic fraction. (**b,c**) Bar graphs represent the relative optical density of TFEB (**b**) and Nrf2 (**c**) localized in nuclei normalized to lamin (A/C). Data shown are mean ± SE of three independent experiments and were analyzed using Student’s *t* test. (**p < 0.01).

**Figure 5:**
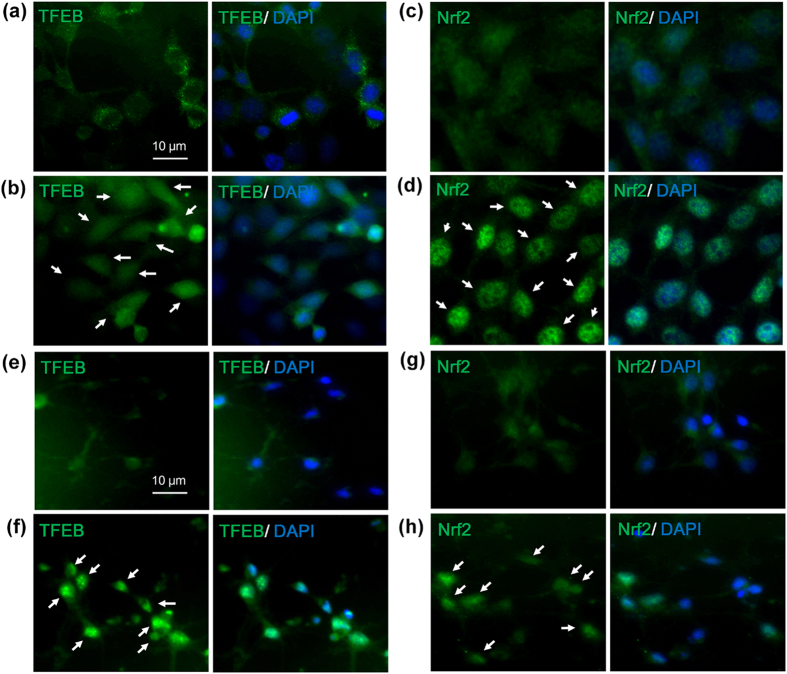
Fisetin increases the levels of TFEB and Nrf2 localized in nuclei. Mouse cortical cells (T4) and rat primary cortical neurons were treated with 5 and 10 μM fisetin for 6 h, respectively. To examine the localization of TFEB and Nrf2, cortical cells and neurons fixed with 4% paraformaldehyde were immunostained using the anti-TFEB and Nrf2 antibodies, respectively. Fluorescence signals were observed using an epifluorescence microscope. (**a,c**) Shows TFEB (**a**) and Nrf2 (**c**) immunosignals in control T4 cells not treated, respectively. (**b,d**) Shows TFEB (**b**) and Nrf2 (**d**) immunosignals in T4 cells treated with 5 μM fisetin, respectively. (**e,g**) Shows TFEB (**e**) and Nrf2 (**g**) immunosignals in control neurons not treated, respectively. (**f,h**) Shows TFEB (**f**) and Nrf2 (**h**) immunosignals in neurons treated with 10 μM fisetin, respectively. Arrows indicate TFEB and Nrf2 localized in the nuclei of T4 cells and primary cortical neurons.

**Figure 6:**
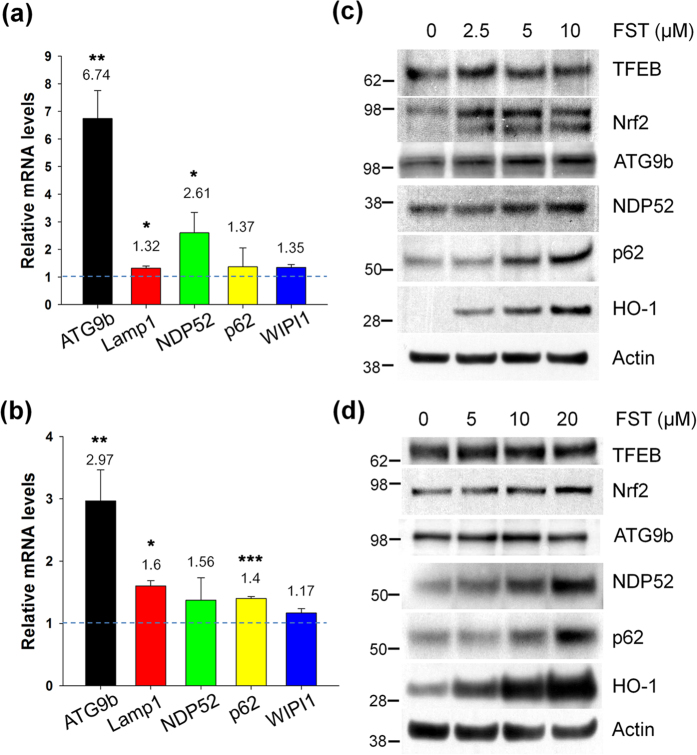
Fisetin induces autophagy and lysosomal genes. (**a,b**) Mouse cortical cells (T4) and rat primary cortical neurons were treated with 5 and 10 μM fisetin for 24 h, respectively. Quantitative real time PCR (qRT-PCR) was performed using primer sets of genes of interest following the procedure described in Methods. Bar graphs represent the relative mRNA level of genes in T4 cells (**a**) and neurons (**b**) compared to those in cells not treated with fisetin. (**c,d**) T4 cells and neurons were treated with fisetin (FST) for 24 h and 36 h, respectively. The levels of TFEB, Nrf2, ATG9b, NDP52, p62/SQSTM1 and HO-1 in T4 cells (**c**) and neurons (**d**) were analyzed by immunoblotting using each corresponding antibody, respectively. Data shown are mean ± SE of three independent experiments and were analyzed using Student’s *t* test. (*p < 0.05; **p < 0.01; ***p < 0.001).

A recent study suggested that the transcriptional factor EB (TFEB) effectively reduces pathological tau species in a tauopathy mouse model^16^. To examine whether TFEB is involved in fisetin-induced decreases of phosphorylated tau, cytosolic and nuclear fractions were prepared from cortical cells treated with vehicle only, 5 or 10 μM of fisetin because upon activation TFEB moves in nucleus^15^. Increases in the level of TFEB were observed in nuclear fractions of cells treated with 5 or 10 μM of fisetin compared to control cells treated with vehicle only (Fig. 4a,b). To further confirm these findings, we carried out immunocytochemical analysis staining for TFEB protein in cortical cells and primary cortical neurons following fisetin treatment to determine its localization. As shown in Fig. 5b,f, increased nuclear localization of TFEB was observed in both cortical cells and primary cortical neurons treated with fisetin, compared to control cells treated with vehicle only. In addition, we analyzed mRNA levels of known TFEB-downstream target genes^14^. As expected, mRNA levels of TFEB-downstream genes such as ATG9b and LAMP1 were significantly increased in both cortical cells and primary neurons treated with fisetin (Fig. 6a,b). Together, these results suggest that fisetin activates TFEB, which might participate in the degradation of phosphorylated tau via the activation of the autophagy-lysosome pathway.

### Fisetin activates Nrf2

We previously showed that the activity of Nrf2 is essential for the clearance of phosphorylated tau via selective autophagy^18^. Fisetin also has been shown to activate Nrf2 in a hippocampal cell line^22^. To examine if fisetin activates Nrf2 in cortical cells, we determined the levels of Nrf2 in the nuclear fractions of control and fisetin treated cells, since activated Nrf2 also moves into the nucleus. As shown in Fig. 4a,c, the levels of Nrf2 in nuclear fractions of cortical cells treated with 5 or 10 μM of fisetin were dramatically increased compared to that of cells not treated. When Nrf2 localization was observed following immunostaining of cortical cells, most of Nrf2 in cells treated with fisetin was nuclear (Fig. 5d). Increased nuclear localization of Nrf2 was also observed in primary cortical neurons treated with fisetin when compared to untreated neurons (Fig. 5h).

The expression of autophagy cargo receptors such as nuclear dot protein 52 (NDP52, also known calcoco2) and p62/SQSTM1 is dependent on Nrf2 activity^18^,^30^. When the transcriptional levels of these genes were examined in cortical cells and primary cortical neurons treated with fisetin, increases in mRNA levels of NDP52 and p62/SQSTM1 were observed in the cells compared to control cells not treated (Fig. 6a,b). As shown in Fig. 6c,d, increased protein levels of the two autophagy receptors along with heme oxygenase (HO)-1, a well-known downstream protein of Nrf2, were also seen in both the cortical cells and primary neurons treated with fisetin. The protein level of p62/SQSTM1 increased by 12 h in cortical cells with fisetin treatment, and then was rapidly decreased, indicating also the activation of autophagy (Supplementary Fig. S3). Together, the results strongly indicate that fisetin is an activator of Nrf2, which could contribute to the clearance of phosphorylated tau via selective autophagy through the induction of its cargo receptors.

### Fisetin induces autophagy

**Figure 7:**
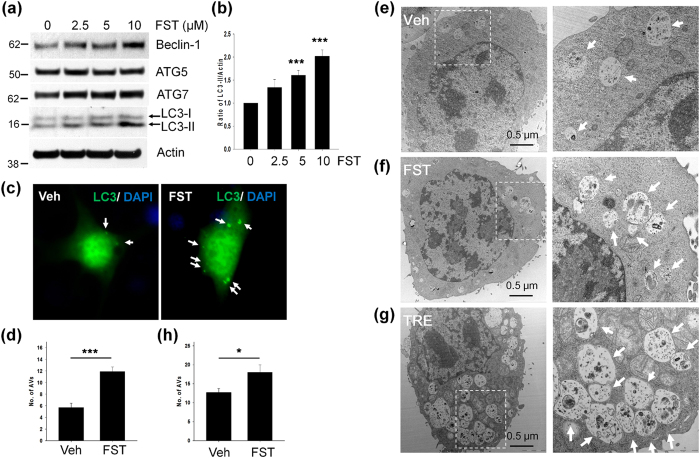
Fisetin activates autophagy. Mouse cortical neuronal cells (T4) were maintained in the presence of doxycycline (1 μg/ml) to induce the expression of tau for 12 h, and subsequently treated with different concentrations of fisetin (FST) for 24 h. (**a**) The levels of beclin-1, ATG5, ATG7 and LC3-II were analyzed by immunoblotting using the anti-beclin-1, ATG5, ATG7 and LC3 antibodies, respectively. (**b**) Bar graphs represent the relative ratio of LC3-II normalized with that of actin. (**c**) The cells transiently transfected with GFP-LC3 were treated with 5 μM FST for 24 h, and observed using an epifluorescence microscope. Arrows indicated autophagic vesicles (AVs). (**d**) Bar graph represents the number of AVs per cell in about 15 cells randomly chosen. (**e–h**) T4 cells were treated with either DMSO (Veh) (**e**), 5 μM fisetin (FST) (**f**) or 100 mM trehalose (TRE) (**g**). (**e–g**) The cell images were taken with transmission electron microscope (TEM). Dotted lines in left panel represent the enlarged area for images on the right, respectively. (**h**) Bar graph represents the number of AVs per cell in approximately 10 cells observed under transmission electron microscope (TEM). Arrows indicate autophagic vesicles (AVs). Data shown are mean ± SE of three independent experiments and were analyzed using Student’s *t* test. (*p < 0.05; ***p < 0.001).

Since fisetin induces genes involved in the autophagy-lysosome pathway (Fig. 6), we speculated that fisetin could induce autophagy itself. To confirm this, we examined the level of LC3-II, a well-known autophagy marker, and autophagy-related gene (ATG) products in mouse cortical cells following treatment of fisetin. As shown in Fig. 7a,b, the levels of LC3-II in cells treated with 5 μM and 10 μM of fisetin were significantly increased along with increases in the amount of ATG proteins including beclin-1 and ATG7 compared to control cells not treated. An increase in the number of autophagic vesicles (AVs) in the cells treated with fisetin (5 μM) was also observed (Fig. 7c–h). When AVs were observed by electron microscopy (EM), the AVs formed in the presence of fisetin had a similar microstructure to those induced by treatment with trehalose, a disaccharide inducer of autophagy (Fig. 7g).

**Figure 8:**
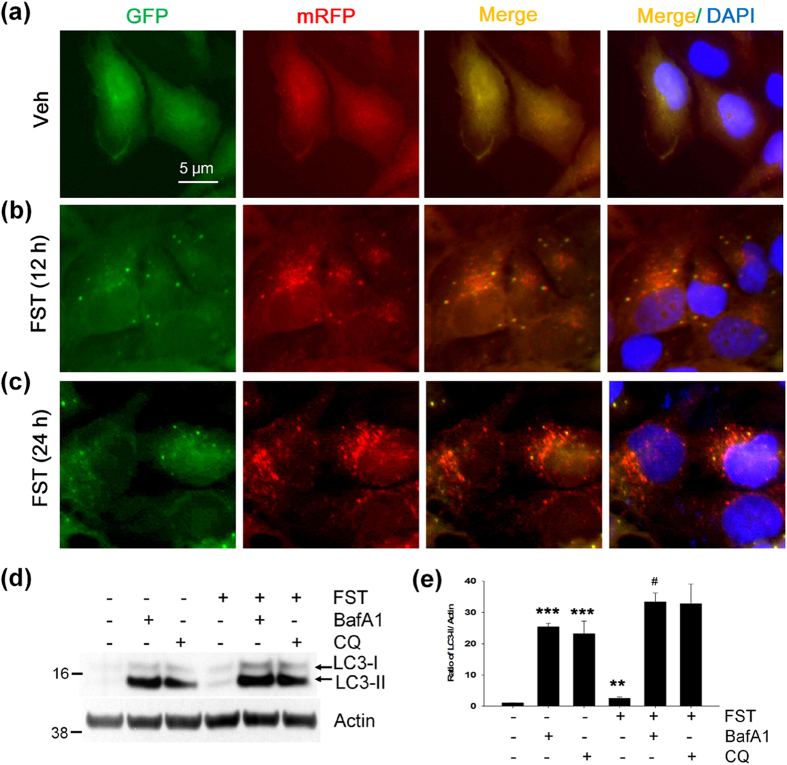
Fisetin increases autophagy flux. (**a–c**) Human neuronal H4 stable cells stably expressing mRFP-GFP-LC3 were treated with either DMSO (Veh) or 10 μM fisetin (FST). The cells were fixed with 4% paraformaldehyde, and fluorescence signals were observed under the epifluorescence microscope. (**d**) Mouse cortical cells (T4) were treated with either DMSO (Veh) or 5 μM fisetin (FST) for 12 h. The cells were then incubated for an additional 18 h following treatment with 100 nM bafilomycin A1 (Baf A1) or 50 μM chloroquine (CQ). The level of LC3-II was analyzed by immunoblotting using the anti-LC3 antibody. (**e**) Bar graphs represent the relative ratio of LC3-II normalized with that of actin. Data shown are mean ± SE of three independent experiments and were analyzed using Student’s *t* test. (**p < 0.01; ***p < 0.001; *cells treated with Baf A1, CQ or fisetin versus cells not treated), (^#^*p* < 0.05; ^#^cells treated with fisetin plus Baf A1 versus cells treated with Baf A1 only).

The increase of LC3-II could represent either autophagy induction or, alternatively, suppression of steps in the autophagy pathway^31^. To further clarify whether the increased levels of LC3-II by fisetin were caused by autophagy induction, we used human neuronal H4 stable cells expressing mRFP-GFP-LC3. As shown in Fig. 8, treatment of the cells with fisetin (10 μM) increased the number of AVs containing LC3, compared to those not treated. Of note, AVs in cells treated with fisetin for 12 h were yellow dots (autophagosomes), whereas most of AVs in cells kept for 24 h were red dots indicative of autophagolysosomes (due to the acidic environment quenching the fluorescence of the GFP), indicating that fisetin induces autophagy and increases its flux. In addition, we treated cortical cells with fisetin, followed by treatment with bafilomycin A1 (Baf A1, 100 nM), an inhibitor of autophagosome-lysosome fusion or chloroquine (CQ, 50 μM), an inhibitor of lysosomal acidification for 18 h. As expected, LC3-II was accumulated in cells treated with Baf A1 or CQ, and the levels of accumulated LC3-II were increased to a greater extent in cells treated with fisetin together with Baf A1 or CQ than those treated with fisetin, Baf A1 or CQ alone (Fig. 8d,e). These results suggest that fisetin induces autophagy via increasing autophagic flux in neuronal cells. Thus, it is likely that fisetin not only stimulates the expression of autophagy cargo receptors, ATGs and lysosomal proteins, but also induces autophagy, which then cooperatively enhances the degradation of phosphorylated tau.

**Figure 9:**
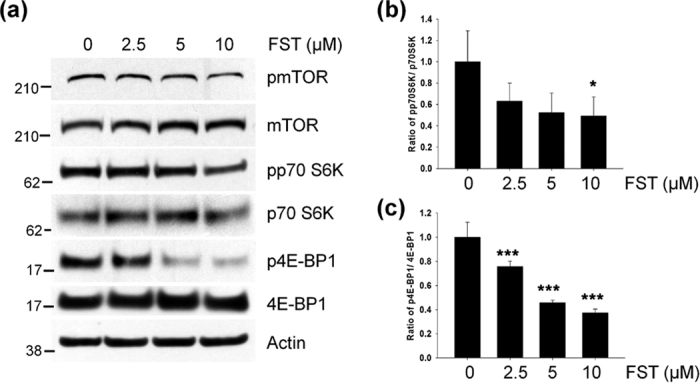
Fisetin inhibits mTOR. (**a**) Mouse cortical cells (T4) were treated with either DMSO or 5 μM fisetin (FST) for 3 h. The levels of mTOR, p70S6K and 4E-BP1 phosphorylation were analyzed by immunoblotting using the anti-pmTOR, pp70S6K and p4E-BP1 antibodies, respectively. (**b,c**) Bar graphs represent the relative ratio of phosphorylated p70S6K (**b**) and 4E-BP1 (**c**) normalized with that of total p70S6K and 4E-BP1, respectively. Data shown are mean ± SE of three independent experiments and were analyzed using Student’s *t* test. (*p < 0.05; ***p < 0.001).

To further understand the molecular mechanism inducing autophagy by fisetin, we analyzed phosphorylation levels of p70S6 kinase and 4E-BP1, downstream proteins of mTORC1 in cortical cells treated with fisetin. As shown in Fig. 9, the phosphorylation levels of 4E-BP1 and p70S6 kinase were significantly decreased in a concentration-dependent manner following treatment with fisetin. Thus, the results indicate that fisetin might not only activate TFEB, but also induce autophagy itself by inhibiting mTORC1 in cortical cells.

### Autophagy plays a crucial role in the degradation of phosphorylated tau

**Figure 10:**
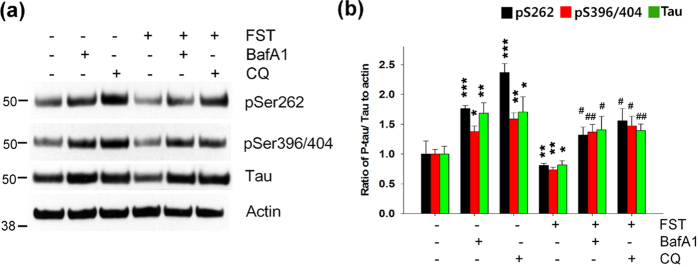
Inhibition of autophagy-lysosome attenuates fisetin-induced degradation of phosphorylated tau. (**a**) Mouse cortical cells (T4) were maintained in the presence of doxycycline (1 μg/ml, Dox) to induce the expression of tau for 12 h, and subsequently treated with either DMSO (Veh) or 5 μM fisetin (FST) for 12 h. The culture media was exchanged for fresh media not containing Dox after washing with PBS. The cells were then incubated for an additional 18 h following treatment with 100 nM bafilomycin A1 (Baf A1) or 50 μM chloroquine (CQ). The levels of tau phosphorylated at Ser262 and Ser396/404 and total tau were analyzed by immunoblotting using the 12E8, PHF1 and tau antibodies, respectively. (**b**) Bar graph represents the relative optical density of phosphorylated tau or tau normalized with that of actin. n = 3. Data shown are mean ± SE and were analyzed using Student’s *t* test. (*p < 0.05; **p < 0.01; ***p < 0.001; *cells treated with Baf A1, CQ or fisetin versus cells not treated), (^#^*p* < 0.05; ^##^*p* < 0.01; ^#^cells treated with fisetin plus Baf A1 or CQ versus cells treated with fisetin only).

Growing evidence indicates that tau is mainly cleared by autophagy^17^,^18^,^19^,^20^. To examine whether decreased levels in phosphorylated tau in neurons treated with fisetin are mediated by fisetin-induced autophagy, we used inhibitors of the autophagy pathway, bafilomycin A1 (Baf A1) and chloroquine (CQ). As expected, treatment of cortical cells with Baf A1 (100 nM) or CQ (50 μM) significantly increased levels of phosphorylated tau as well as total tau. Of note, these inhibitors significantly hindered fisetin-induced degradation of phosphorylated tau (Fig. 10a,b). Thus, these data strongly indicate that fisetin-induced autophagy plays a major role in the degradation of phosphorylated tau.

### Knockdown of TFEB or Nrf2 attenuates fisetin-induced degradation of phosphorylated tau

**Figure 11:**
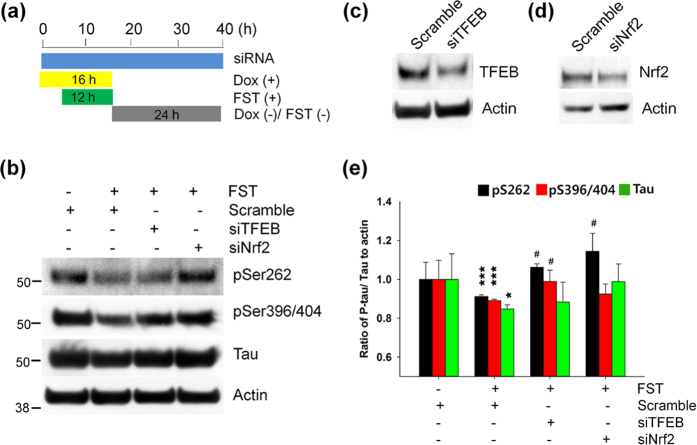
Knockdown of TFEB or Nrf2 attenuates the clearance of phosphorylated tau by fisetin treatment. (**a**) Mouse cortical cells (T4) were transiently transfected with either siRNA or scramble RNA as a control and maintained in the presence of doxycycline (1 μg/ml, Dox) to induce the expression of tau. The cells were treated with 5 μM fisetin (FST) for 12 h. The culture media was exchanged for fresh media not containing Dox after washing with PBS. The cells were then incubated for an additional 24 h according to the experiment scheme shown as a diagram. (**b**) The levels of tau phosphorylated at Ser262 and Ser396/404, and total tau were analyzed by immunoblotting using the 12E8, PHF1 and tau antibodies, respectively. (**c,d**) In T4 cells transfected with either siRNA ((**c)** TFEB; (**d)** Nrf2) or scramble RNA as a control, the levels of TFEB and Nrf2 were analyzed by immunoblotting using anti-TFEB and Nrf2 antibodies, respectively. (**e**) Bar graph represents the relative optical density of phosphorylated tau or tau normalized with that of actin. n = 3. Data shown are mean ± SE and were analyzed using Student’s *t* test. (*p < 0.05; ***p < 0.001; *cells treated with fisetin versus cells not treated), (^#^*p* < 0.05; ^#^cells siRNA transfected versus cells scramble RNA transfected in the presence of fisetin).

To further demonstrate that TFEB and Nrf2 play essential roles in fisetin-induced phosphorylated tau degradation, we used siRNA specific to each gene to knockdown gene expression (Fig. 11c,d). As shown in Fig. 11b,e, fisetin reduced the levels of phosphorylated tau; by contrast, the levels in cortical cells transfected with siRNA specific to TFEB or Nrf2 were similar to the level in cells not treated with fisetin. The similar results were observed in cortical cells transfected with siRNA specific to NDP52 or p62/SQSTM1 (Supplementary Fig. S4). These results support the conclusion that fisetin-induced phosphorylated tau decrease is highly dependent on the activation of TFEB and Nrf2, and occurs via selective autophagy by its cargo receptors.

## Discussion

**Figure 12:**
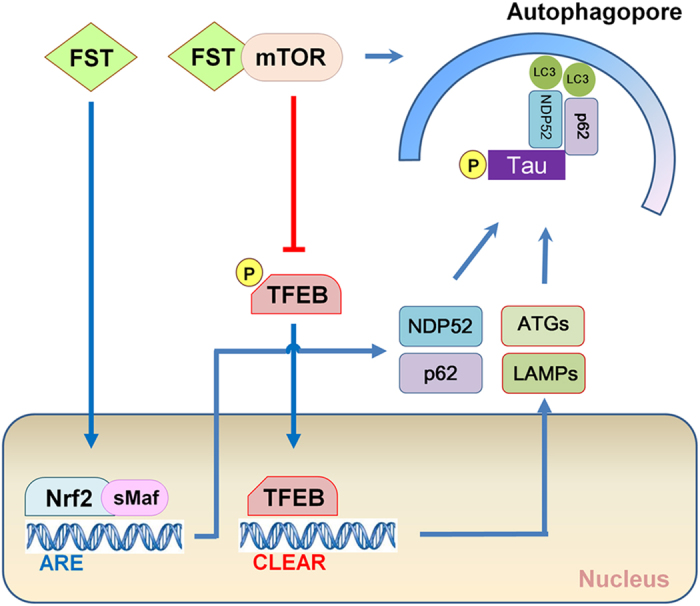
Schematic representation of the effect of fisetin on the clearance of phosphorylated tau.

Tau, a microtubule-associated neuronal protein, becomes inappropriately phosphorylated and eventually accumulates as NFTs in AD brain^32^,^33^. Increasing evidence indicates that pathologically phosphorylated tau and its oligomerized forms are toxic, compromising neuronal function^34^,^35^. Therefore, the strategy of promoting the degradation of pathological tau in AD has been suggested as a therapeutic approach^33^,^36^,^37^. In this study, we show for the first time that fisetin, a small flavonoid molecule, stimulates the degradation of phosphorylated tau via the activation of TFEB and Nrf2 which elicits the expression of autophagy and lysosomal genes (Fig. 12).

TFEB is known to be as a master regulator of the autophagy and lysosome pathway due to its ability to increase the expression of autophagy and lysosomal genes, and promote lysosomal biogenesis^13^,^14^. Given the accumulating evidence indicates that tau is degraded via autophagy^17^,^18^,^19^,^20^, it was expected that induction of TFEB could enhance the clearance of phosphorylated tau in neurons. A recent study provided a notable result that TFEB effectively does not only reduce the levels of aberrant tau, but also improve cognitive function in a tauopathy mouse model^16^. In the study, they proposed that small molecules which activate TFEB could be beneficial in AD. Here, we show that fisetin activates TFEB (Fig. 4, 5, 6), and provide novel evidence that in primary cortical neurons as well as cortical cells fisetin stimulates the degradation of phosphorylated tau via the activation of TFEB (Figs 1 and 11). A recent study reported that TFEB accelerates lysosomal degradation of amyloid precursor protein (APP), thus reducing Aβ generation and amyloid plaque pathogenesis^38^. Thus, it is suggested that fisetin should be evaluated further as a possible therapeutic agent for AD.

Nrf2 is a key regulator of cellular resistance against oxidants^39^. Previously, it has been shown that in immortalized mouse hippocampal HT22 cells fisetin was able to activate Nrf2 and ATF4, resulting in increased levels of glutathione (GSH) and protection against oxidative stress-induced damage^22^. In this study we showed that in a neuronal model and in primary neuron fisetin activates Nrf2 (Figs 4 and 5), which is linked to the enhanced expression of autophagy receptor, NDP52, thus facilitating the degradation of phosphorylated tau species through the selective autophagy^18^ (Figs 6 and 12). p62/SQSTM1 also likely plays a role in tau degradation as phosphorylated tau levels are increased in p62/SQSTM1 knockout mice^40^. Since the expression of p62/SQSTM1 is also highly dependent upon Nrf2 activity^30^, it can be suggested that the induction of Nrf2 together with TFEB by fisetin provides an optimal condition for facilitating the degradation of phosphorylated tau. Also, knockdown of either TFEB or Nrf2 attenuated fisetin-mediated reduction in phosphorylated tau levels (Fig. 11), thus indicating each transcription factor plays a crucial role in the degradation of phosphorylated tau.

The kinase mTOR is a component of two mTOR complexes, mTORC1 and mTORC2. mTORC1 is a downstream target of the phosphatidylinositol 3 kinase (PI3K)/AKT pathway, and is well known as a negative modulator of autophagy^41^. The activation of mTORC1 leads to the phosphorylation of numerous target proteins such as p70S6K and 4E-BP1, which participate in protein translation, ribosomal biogenesis and cell growth^42^. Conversely, the inhibition of mTORC1 by starvation or stressors induces autophagy in part via dephosphorylation of autophagy/beclin-1 regulator (AMBRA1) and activation of unc-51 like autophagy activating kinase 1 (ULK1)^43^. Recently, it was shown that mTORC1 associated with the cytosolic surface of lysosomes directly phosphorylates TFEB, thus sequestering it in the cytoplasm and hindering its activation^15^. When mTORC1 activity was inhibited, TFEB moves into the nucleus, thus inducing the expression of downstream genes^14^,^15^. Considering that the clearance of overexpressed, hyperphosphorylated and misfolded tau was mediated via TFEB activation in a mouse tauopathy model^16^, it is speculated that the inhibiting mTORC1 could be a potential therapeutic strategy for increasing the degradation of pathological tau species in AD. Interestingly, some studies have suggested that fisetin inhibits mTOR activity through direct binding^23^,^24^. In our study, fisetin treatment also reduced the phosphorylation levels of p70S6K and 4E-BP1 (Fig. 9), reflecting the inhibitory effect of fisetin on mTORC1 activity. Taken together it is likely that fisetin-induced activation of TFEB (Figs 4 and 6) and autophagy (Figs 7 and 8) may be due to its ability to directly inhibit mTORC1.

Interestingly, in the study in which adenovirus was used to express TFEB in the brains of the tauopathy mice only the levels of the exogenously expressed, phosphorylated tau species were reduced, endogenous tau levels were unaffected^16^. This is in contrast to the present study where fisetin-treatment of a neuronal model resulted in a decrease in the levels of non-pathological tau (Fig. 10). It can be speculated that this may be due to the fact that fisetin upregulates the activity of Nrf2 in addition to TFEB, and both are required for facilitating the clearance of endogenous, soluble tau species. Recently, studies using mice and neurons devoid of tau suggest that partial reduction of overall tau levels could effectively protect neurons against Aβ and excitotoxicity^3^,^4^,^5^,^6^,^35^, demonstrating that fisetin-mediated decrease in total tau level might be beneficial for AD patients. Overall the results of this study strongly indicate that fisetin may be a good candidate molecule for facilitating autophagy and aberrant tau degradation in the content of AD and other tauopathies.

## Methods

### Antibodies and reagents

PHF1 (phospho-Ser396/404) and 12E8 (phospho-Ser262) antibodies were kindly provided by Dr. P. Davies and Dr. P. Seubert, respectively. Anti-tau polyclonal (A0024) antibody was purchased from Dako. Anti-Nrf2 (12721), TFEB (4220), mTOR (2983), pmTOR (phospho-Ser2448, 5536), p70S6K (2708), pp70S6K (phospho-Thr389, 9234), 4E-BP1 (9644), p4E-BP1 (phospho-Thr37/46, 2855), NDP52 (9036) and HA (2367) antibodies were purchased from Cell Signaling Technology. Anti-LC3 antibody (PD014) was obtained from Medical & Biological Laboratories. Anti-lamin (A/C) (SC-6215) and β-actin (MAB1501) antibodies were purchased from Santa Cruz Biotechnology and Millipore, respectively. Anti-heme oxygenase (HO)-1 (ADI-SPA-895) and p62/SQSTM1 (BML-PW9860) antibodies were obtained from Enzo Life Sciences. Anti-ATG9b (PA5-20998) antibody was purchased from Thermo Fisher Scientific. Anti-tubulin (T6074) antibody was obtained from Sigma. The GFP-LC3 plasmid was used previously^18^. The GSK-3β-S9A plasmid was kindly provided by Dr. E. Choi. The mouse TFEB specific predesigned siRNA (S74859) was purchased from Life Technology. The ON-TARGET plus mouse Nrf2 (18024) siRNA SMART pool was purchased from GE Healthcare. Fisetin (5016) was purchased from Tocris. Chemicals such as bafilomycin A1 (B1793) and chloroquine (C6628) were obtained from Sigma.

### Cell culture

Immortalized mouse cortical neuronal cells (T4) engineered to inducibly express full-length tau in the presence of the tetracycline derivative doxycycline have been previously described^44^. The cells were cultured in Dulbecco’s modified Eagle’s medium (DMEM) supplemented with 10% fetal bovine serum (FBS), 0.1% gentamicin, 4 mM glutamine, and 10 units/ml penicillin and 100 units/ml streptomycin at 33 °C in a humidified atmosphere containing 5% CO_2_. In this study, cells were treated with doxycycline at a concentration of 1 μg/ml for 24 h to induce tau expression. The human neuronal H4 stable cell line stably expressing mRFP-GFP-LC3 was previously described^45^. HEK 293 cells were cultured in the same medium with T4 cells at 37 °C in 5% CO_2_.

### Primary cell culture

Primary cortical neuronal cultures from rat embryonic forebrain were prepared and maintained as described previously with modification^46^. In brief, whole brains were removed from E18 rats. The cortices were dissected, treated with 0.05% trypsin at 37 °C for 30 min, and gently triturated with a fire polished glass Pasteur pipette. Dissociated cells were plated onto 6-well plates or glass-cover slips in 12-well plates coated with poly-D-lysine (40 μg/ml, Millipore, A-003-E) in MEM (Invitrogen, 11090) containing 5% FBS, 20 mM glucose, 2 mM glutamine in the incubator at 37 °C in a humidified atmosphere containing 5% CO_2_. Five hours after plating, medium was replaced with Neurobasal medium (NBM, Invitrogen, 21103–049) supplemented with 0.5 mM glutamine and 2% B27 (Invitrogen, 17504–044). One-half of the medium was replaced with the fresh, complete NBM every 3 days. Experiments were performed on DIV 10–12.

### Protein kinase assay

Mouse cortical neuronal cells (T4) were lysed in lysis buffer (50 mM Tris-HCl [pH 7.9], 100 mM NaCl, 10 mM MgCl_2_, 1 mM DTT) containing 0.1% NP-40 and protease inhibitors. Proteins were extracted on ice with periodic vortexing for 30 min, and lysates were cleared by centrifugation at 10,000 × *g* for 10 min at 4 °C. Ten micrograms of the lysates was incubated with 2 μg of GST-tau protein at 32 °C for 40 min in a kinase reaction buffer (Cell Signaling Technology) containing 10 μM cAMP (Tocris), 5 nM okadaic acid (Cell Signaling Technology) and 1 mM ATP (Sigma). The reaction was stopped by the addition of 1x SDS-sample loading buffer and incubation at 95 °C for 5 min. Protein was separated by SDS-PAGE and immunoblotted as described below.

### Phosphatase assay

Phosphatase activity of mouse cortical neuronal cells (T4) was quantitated using the Serine/Threonine Phosphatase Assay System (Promega, V2460) by measuring the dephosphorylation of a phospho-peptide, RRA(pT)VA. Briefly, T4 cells were lysed in the phosphatase storage buffer (10 mM Tris-HCl [pH 7.4], 150 mM NaCl, 1 mM EDTA, 1 mM EGTA, 0.5% NP-40) containing protease inhibitors. Proteins were extracted on ice with periodic vortexing for 30 min, and lysates were cleared by centrifugation at 10,000 × *g* for 10 min at 4 °C. Two hundred microliters of supernatants was passed through Sephadex G-25 column to remove the residual phosphate in the supernatants. Two microliters of the samples was incubated with 100 μM of phosphopeptide (RRA(pT)VA) at 37 °C for 30 min in buffer consisting of 50 mM imidazole [pH 7.2], 0.2 mM EGTA, 0.1 mg/ml BSA and 0.02% β-mercaptoethanol without or with 20 nM okadaic acid. To terminate the reaction, 50 μl of molybdate dye with additive mixture was added to the mixture. The mixture was incubated for 10 min at room temperature to allow color development. Absorbance was measured at 600 nm using the SpectraMax M microplate reader (Molecular Devices). Phosphate released was determined by comparing absorbance to a standard phosphate curve.

### Immunoblotting

Mouse cortical neuronal cells (T4) and primary cortical neurons were washed once with PBS and lysed with modified RIPA buffer (10 mM Tris-HCl [pH 7.4], 150 mM NaCl, 1 mM EGTA, 1% NP-40, 0.25% sodium deoxycholate, 0.1% SDS) containing 1 mM NaF, 1 mM Na_3_VO_4_, 1 mM PMSF and 10 μg/ml each of aprotinin, leupeptin and pepstatin. Proteins were extracted on ice with periodic vortexing for 30–40 min, and lysates were cleared by centrifugation at 10,000 × *g* for 10 min at 4 °C, and the supernatants were used for immunoblotting following boiling in 1x SDS-sample loading buffer for 5 min. For analysis protein samples (40 μg) were separated on Bolt® 4–12% Bis-Tris Plus gradient gels (Invitrogen) at a constant current of 20 mA followed by transfer to nitrocellulose membranes (GE Healthcare) and immunoblotting with the indicated antibodies. Blots were developed with chemiluminescence (GE Healthcare). All protein concentrations were determined using the BCA method (Sigma).

### Sarkosyl fractionation assay

The assay was carried out as described previously with a few modifications^25^,^26^. HEK 293 cells were lysed with modified RIPA buffer containing 1 mM NaF, 1 mM Na_3_VO_4_, 1 mM PMSF and 10 μg/ml each of aprotinin, leupeptin and pepstatin. Proteins were extracted on ice with periodic vortexing for 30 min, and lysates were cleared by centrifugation at 10,000 × *g* for 10 min at 4 °C, and the supernatants were used for RIPA-soluble fractions. To prepare the sarkosyl-insoluble fraction, the pellets were resuspended in RAB buffer (100 mM Mes [pH6.8], 2 mM EGTA, 0.5 mM MgSO_4_, 500 mM NaCl, 1 mM MgCl_2_, 10 mM NaH_2_PO_4_, 20 mM NaF) containing 0.5% N-lauroylsarcosine (Sarkosyl) with protease inhibitors, vortexed for 1 min at room temperature, incubated at 4 °C overnight, and then centrifuged at 16,000 × g for 30 min at room temperature. The supernatants were collected as sarkosyl-soluble fractions, and the pellets, sarkosyl-insoluble fractions, were resuspended in 1× SDS protein loading buffer and incubated in 95 °C for 5 min. Samples were electrophoresed on Bolt® 4–12% Bis-Tris Plus gradient gels and immunoblotted for tau as described above.

### Quantitative real-time PCR

For the synthesis of cDNA, total RNA was isolated using Trizol (Invitrogen) according to the manufacture’s protocol. cDNA was generated using 2 μg of RNA by the use of RT-PCR EcoDry premix (Clontech). Quantitative real-time PCR (qRT-PCR) was performed using the SYBR® Green real-time PCR master mix (Invitrogen, 4344463) on a Real-Time PCR Detection System (Applied Biosystems®, 7500). Each reaction consisted of 10 μl of cDNA product from the diluted reverse transcription reaction (×35), 2.5 μM of primers (mouse ATG9b, 5′-AGCTATCATCAGCGGAATGG-3′ and 5′-GCGAAGGAGGAAGGTTGTAA-3′; mouse LAMP1, 5′-GACCCTGAAAGTGGAGAACA A-3′ and 5′-GGGCATCAGGAAGAGTCATATT-3′; mouse NDP52, 5′-TGGCAACTTCTC TCAGGTCCTGTT-3′ and 5′-TCCTTGCGTCGAGGGATGAACTTT-3′; mouse p62, 5′-GTGGTGGGAACTCGCTATAAG-3′ and 5′-GAAAGATGAGCTTGCTGTGTTC-3′; mouse WIPI1, 5′-CACAGGATGGAGGAGAATGTG-3′ and 5′-TGCAGCATAAGATGGAGGTA AG-3′; mouse GAPDH, 5′-TCAACAGCAACTCCCACTCTTCCA-3′ and 5′-ACCCTGTTG CTGTAGCCGTATTCA-3′; rat ATG9b, 5′-TCTGTTGGTCTTCCTCCTAGT-3′ and 5′-GTCCCAGTAGCTGAAGAGATTG-3′; rat LAMP1, 5′-GCACTGTTCGAGGTGAAAGA-3′ and 5′-CAGCATCATAGGTGGTCAGAAA-3′; rat NDP52, 5′-GGAGACGACCCAGGA GTATTA-3′ and 5′-CTCTTCATCCTTGGGCAGATAG-3′; rat p62, 5′-CTAGGCATCGAGG TTGACATT-3′ and 5′-CTTGGCTGAGTACCACTCTTATC-3′; rat WIPI1, 5′-GCCCTT TCAATCAACCATTCC-3′ and 5′-TTTCAGGGAGTTTCCGTCATAG-3′; rat GAPDH, 5′-ACTCCCATTCTTCCACCTTTG-3′ and 5′-CCCTGTTGC TGTAGCCATATT-3′), and 12.5 μl of SYBR® Green real-time PCR master mix. The reactions were incubated in a 96-well plate at 95 °C for 5 min, followed by 40 cycles of 95 °C for 15 s, 61 °C for 20 s and 72 °C for 40 s. After the reactions were completed, the threshold was manually set and the threshold cycle (CT) was automatically recorded. The CT is defined as the fractional cycle number at which the fluorescence signal passes the fixed threshold. All reactions were run in three replicates for each sample.

### Electron microscopy (EM)

Mouse cortical neuronal cells (T4) were treated with 5 μM fisetin for 24 h or 100 mM trehalose for 12 h. Cells were fixed overnight in a mixture of cold 2.5% glutaraldehyde in 0.1 M phosphate buffer (pH 7.2) and 2% paraformaldehyde (PFA) in 0.1 M phosphate buffer (pH 7.2) and embedded with epoxy resin. The epoxy resin-mixed samples were loaded into capsules and polymerized at 60 °C for 48 h. Thin sections were sliced on an ultramicrotome (Leica, UC7) and collected on a copper grid. Appropriate areas for thin sectioning were cut at 70 nm and stained with saturated 2% uranyl acetate and 2% lead citrate before examination on a transmission electron microscope (Carl Zeiss, Libra 120) at 120 kV.

### Immunohistochemical staining

Mouse cortical neuronal cells (T4) and primary cortical neurons were fixed with 4% paraformaldehyde for 20 min. The cells were permeabilized by incubation in 0.2% Triton-X-100 in PBS for 30 min and blocked in a PBS blocking solution (2% BSA, 0.1% Triton-X-100 in PBS) for 1 h following a short washing with PBS. The cells were incubated with the appropriate primary antibodies (rabbit anti-TFEB [1:250] or anti-Nrf2 [1:250]) diluted in the blocking solution at 4 °C overnight. Then, the cells were incubated with donkey anti-rabbit Alexa488 conjugated antibodies (1:500) for 1 h at room temperature. The cover-slips were mounted on the glass slides with ProLong® gold antifade reagent (Invitrogen, P36935) following three washes with PBS. Images were photographed using a fluorescence microscope (Carl Zeiss).

### Preparation of nuclear and cytosolic fractions

Mouse cortical neuronal cells (T4) were washed with and scraped in PBS. Cell pellets were resuspended in fractionation buffer (10 mM HEPES [pH 7.9], 10 mM KCl, 1.5 mM MgCl_2_, 0.1% NP-40, 0.5 mM NaF, 200 mM Na_3_VO_4_ and 1 × protease inhibitor cocktail). The cells were incubated on ice for 15 min with shaking. Lysates were centrifuged at 2,600 × g at 4 °C, and supernatants representing cytosolic fraction were collected. Subsequent to washing precipitates using the fractionation buffer without 0.1% NP-40, the precipitates then were resuspended with the modified RIPA buffer containing 1x protease inhibitor cocktail and incubated on ice for 20 min with periodic vortexing. The lysates were then cleared by centrifugation at 10,000 × g at 4 °C, and supernatants were used as the nucleic fractions.

### siRNA transfection

For knockdown of endogenous genes, specific siRNA or scrambled RNA as a control was mixed with Oligofectamine (Invitrogen, 12252–011) in serum-free OPTI-MEM medium for 10 min. The mixture was loaded onto T4 cortical neuronal cells following the exchange of culture medium for serum-free DMEM medium. After 4 h, FBS was added up to 5% and cells were maintained at 33 °C in a humidified atmosphere containing 5% CO_2_.

## Additional Information

**How to cite this article**: Kim, S. *et al*. Fisetin stimulates autophagic degradation of phosphorylated tau via the activation of TFEB and Nrf2 transcription factors. *Sci. Rep*. **6**, 24933; doi: 10.1038/srep24933 (2016).

## Supplementary Material

Supplementary Information
