# Supplementary material for: Fisetin stimulates autophagic degradation of phosphorylated tau via the activation of TFEB and Nrf2 transcription factors
Source: Sci Rep. 2016 Apr 26;6:24933. doi: 10.1038/srep24933 (PMC4844953; doi:10.1038/srep24933)
Supplement: Supplementary Information [file srep24933-s1.pdf]

Supplementary Information

Fisetin stimulates autophagic degradation of phosphorylated tau via the activation of TFEB and Nrf2 transcription factors

Sunhyo Kim[1], Ki Ju Choi[2], Sun-Jung Cho[1], Sang-Moon Yun[1], Jae-Pil Jeon[1], Young Ho Koh[1], Jihyun Song[1], Gail V.W. Johnson[3] and Chulman Jo[1*]

Supplementary Method

Antibodies and reagents. Anti-pGSK-3β (Ser9, 9336) and GSK-3β (9315) antibodies were purchased from Cell Signaling Technology. Anti-pERK1/2 (SC-7383) and ERK1/2 (SC-94) antibodies were purchased from Santa Cruz Biotechnology. The mouse p62/SQSTM1 specific predesigned siRNA (6399) was purchased from Cell Signaling Technology. The mouse NDP52 specific siRNA (141979) was purchased from Santa Cruz Biotechnology.

MTT assay. *C*ell viability was assessed by using thiazolyl blue tetrazolium bromide (MTT) dye as a colorimetric assay. Mouse cortical (T4) cells and primary neurons with fisetin (FST) grown in 24 well plates were treated with fisetin for 24 h and 36 h, respectively, MTT solution (5 mg/ml) in PBS was added to the media. Two hour after incubation, the media was removed, dimethyl sulfoxide (DMSO) added to each well and the cells resuspended. The fluorescence was measured using the SpectraMax M microplate reader (Molecular Devices) with excitation at 560 nm and background at 670 nm. Results are presented as a percent of control cells.

Supplementary Figure S1

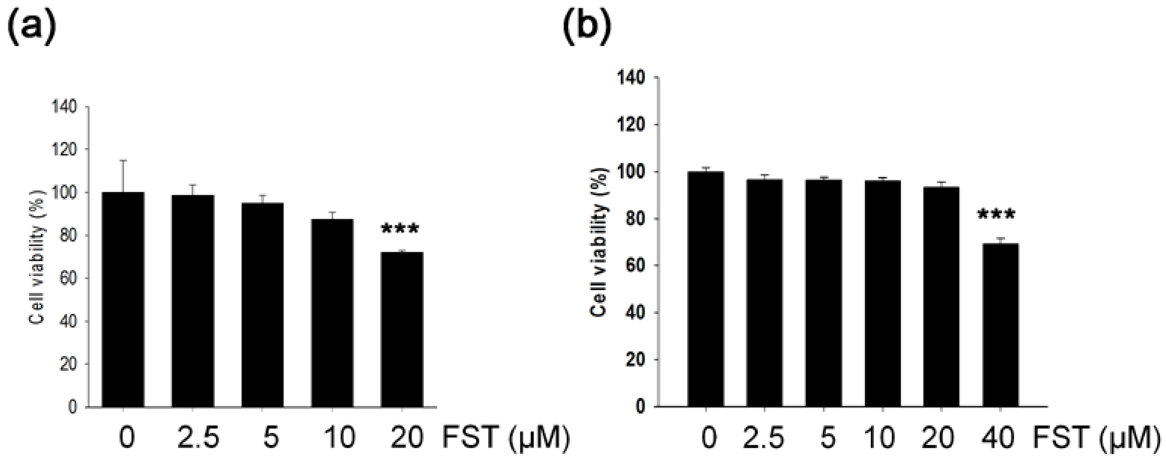

Supplementary Figure S1. Determination of appropriate concentration of fisetin used in the experiment. Mouse cortical cells (T4 cells) (**a**) and primary cultured cortical neurons (**b**) were treated with the indicated concentration of fisetin (FST) for 24 h and 36 h, respectively. The cell viability was examined by the MTT assay. Data shown are mean ± SE and were analyzed using Student's *t* test. (***, p<0.001)

Supplementary Figure S2

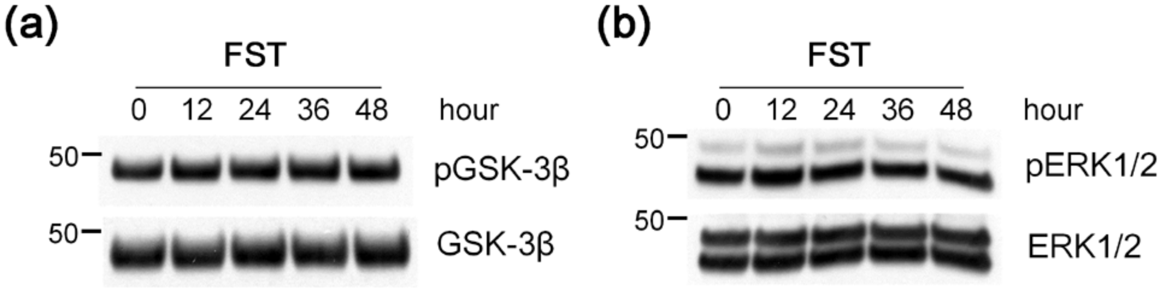

Supplementary Figure S2. The phosphorylation levels of GSK-3β and ERK1/2 in the presence of fisetin. (**a, b**) Mouse cortical neuronal cells (T4) were maintained in the presence of doxycycline (1 ug/ml) to induce the expression of tau for 12 h, and subsequently treated with 5 μM fisetin. The cells were kept in CO_2 incubator during indicated time. Then, the levels of phosphorylated GSK-3β (**a**) and ERK1/2 (**b**) were analyzed by immunoblotting using the phospho-specific GSK-3β and ERK1/2 antibodies, respectively.

Supplementary Figure S3

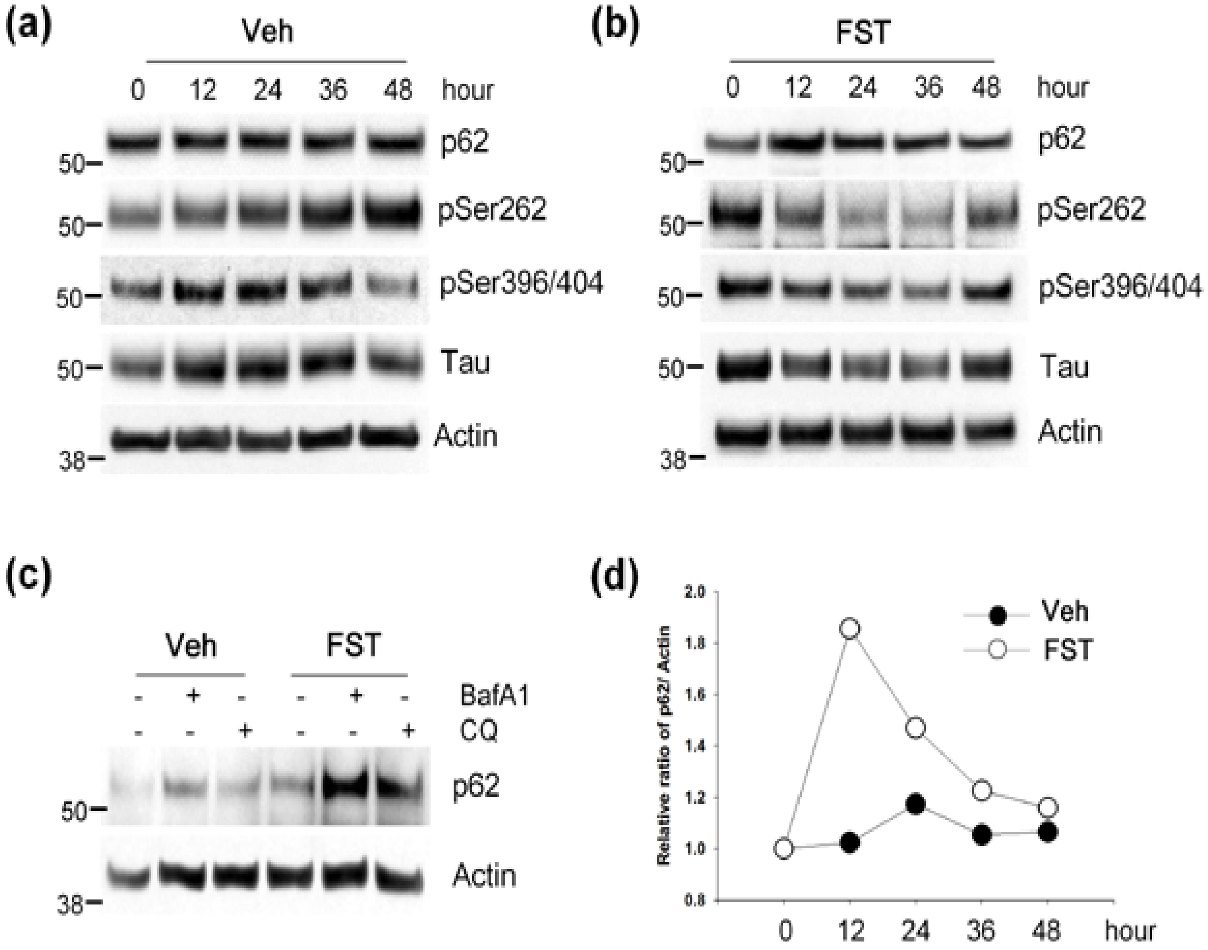

Supplementary Figure S3. The levels of p62/SQSTM1 protein in the presence of fisetin. (**a, b**) Mouse cortical neuronal cells (T4) were maintained in the presence of doxycycline (1 ug/ml) to induce the expression of tau for 12 h, and subsequently treated with either DMSO (**a**, Veh) or 5 μM fisetin (**b**, FST). The cells were kept in CO_2 incubator during indicated time. Then, cell lysates were analyzed by immunoblotting using the anti-p62/SQSTM1 antibody. The levels of tau phosphorylated at Ser262 and Ser396/404, and total tau were analyzed by immunoblotting using the 12E8, PHF1 and tau antibodies, respectively. (**c**) T4 cells were treated with either DMSO (Veh) or 5 μM fisetin (FST) for 12 h. The culture media was exchanged for fresh media not containing FST after washing with PBS. The cells were then incubated for an additional 18 h following treatment with 100 nM bafilomycin A1 (Baf A1) or 50 μM chloroquine (CQ). The levels of p62/SQSTM1 were analyzed by immunoblotting using an anti-p62/SQSTM1 antibody. (**d**) Graph represents the relative optical density of p62 normalized with that of actin.

Supplementary Figure S4

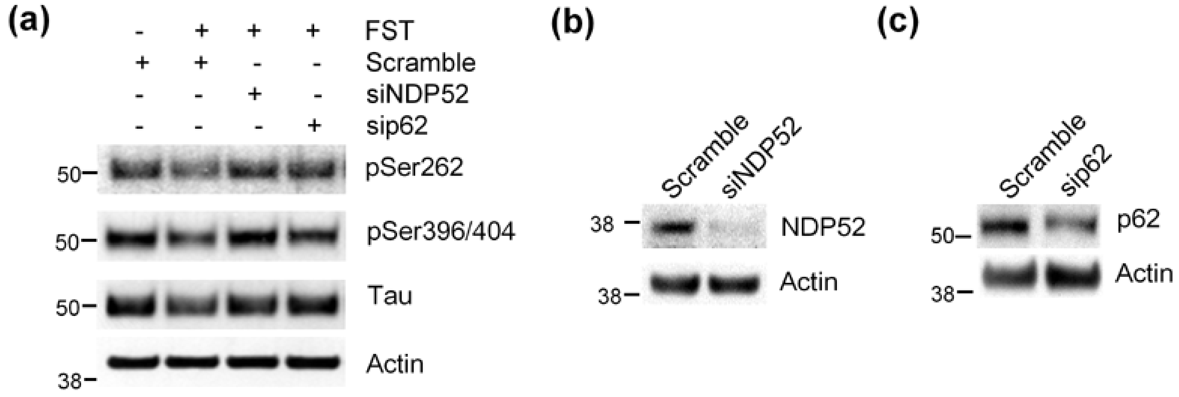

Supplementary Figure S4. Knockdown of NDP52 or p62/SQSTM1 attenuates the clearance of phosphorylated tau by fisetin treatment. (**a**) Mouse cortical cells (T4) were transiently transfected with either siRNA or scramble RNA as a control and maintained in the presence of doxycycline (1 μg/ml, Dox) to induce the expression of tau. The cells were treated with 5 μM fisetin (FST) for 12 h. The culture media was exchanged for fresh media not containing Dox after washing with PBS. The cells were then incubated for an additional 24 h according to the experiment scheme shown as a diagram. The levels of tau phosphorylated at Ser262 and Ser396/404, and total tau were analyzed by immunoblotting using the 12E8, PHF1 and tau antibodies, respectively. (**b, c**) In T4 cells transfected with either siRNA (**b**, NDP52; **c**, p62/SQSTM1) or scramble RNA as a control, the levels of NDP52 and p62/SQSTM1 were analyzed by immunoblotting using anti- NDP52 and p62/SQSTM1 antibodies, respectively.
